# The Effects of Hypoxic Preconditioned Murine Mesenchymal Stem Cells on Post-Infarct Arrhythmias in the Mouse Model

**DOI:** 10.3390/ijms23168843

**Published:** 2022-08-09

**Authors:** Beschan Ahmad, Anna Skorska, Markus Wolfien, Haval Sadraddin, Heiko Lemcke, Praveen Vasudevan, Olaf Wolkenhauer, Gustav Steinhoff, Robert David, Ralf Gaebel

**Affiliations:** 1 Department of Cardiac Surgery, Rostock University Medical Center, 18057 Rostock, Germany; 2 Department of Life, Light & Matter, Interdisciplinary Faculty, Rostock University, 18059 Rostock, Germany; 3 Department of Systems Biology and Bioinformatics, University of Rostock, 18051 Rostock, Germany; 4 Institute for Medical Informatics and Biometry, Faculty of Medicine Carl Gustav Carus, Technical University Dresden, 01307 Dresden, Germany

**Keywords:** mesenchymal stem cells, myocardial infarction, calcium regulation, ventricular arrhythmia, hypoxic precondition

## Abstract

Ventricular arrhythmias associated with myocardial infarction (MI) have a significant impact on mortality in patients following heart attack. Therefore, targeted reduction of arrhythmia represents a therapeutic approach for the prevention and treatment of severe events after infarction. Recent research transplanting mesenchymal stem cells (MSC) showed their potential in MI therapy. Our study aimed to investigate the effects of MSC injection on post-infarction arrhythmia. We used our murine double infarction model, which we previously established, to more closely mimic the clinical situation and intramyocardially injected hypoxic pre-conditioned murine MSC to the infarction border. Thereafter, various types of arrhythmias were recorded and analyzed. We observed a homogenous distribution of all types of arrhythmias after the first infarction, without any significant differences between the groups. Yet, MSC therapy after double infarction led to a highly significant reduction in simple and complex arrhythmias. Moreover, RNA-sequencing of samples from stem cell treated mice after re-infarction demonstrated a significant decline in most arrhythmias with reduced inflammatory pathways. Additionally, following stem-cell therapy we found numerous highly expressed genes to be either linked to lowering the risk of heart failure, cardiomyopathy or sudden cardiac death. Moreover, genes known to be associated with arrhythmogenesis and key mutations underlying arrhythmias were downregulated. In summary, our stem-cell therapy led to a reduction in cardiac arrhythmias after MI and showed a downregulation of already established inflammatory pathways. Furthermore, our study reveals gene regulation pathways that have a potentially direct influence on arrhythmogenesis after myocardial infarction.

## 1. Introduction

Chronic ischemic heart disease subsequent to a myocardial infarction (MI) is one of the leading causes of death in western countries [1]. High blood pressure, low density lipoprotein, diabetes mellitus, nicotine abuse, or family predispositions are the most common risk factors for severe cardiovascular events. In the majority of cases, MI is followed by ruptured arteriosclerotic plaques, causing hypoxic ischemia in the coronary artery. As a result, macrophages and granulocytes migrate in order to activate the sympathetic and humoral immune system [2], and further remodeling processes are often observed in the infarct area, the border zone, and healthy heart tissue [3]. These factors lead to a higher mortality of 10–15% in patients, two years after infarction. A major part of it is attributed to cardiac adverse events [4] and half of these are associated with cardiac arrhythmias [5].

In addition to advances in MI therapy, methods for improving prognostic outcomes are limited. More recent studies on MI therapies showed that mesenchymal stem cells (MSC) have a high potential for positive hemodynamic, cell biological, and electrophysiological effects [6,7,8]. MSC are characterized by adherent culture in vitro, specific antigen expression, and multipotent properties [9]. The purity and proliferation of MSC culture are of great importance. Mimicking the physiological environment in vivo by culturing MSC under conditions of low oxygen concentration is crucial for efficient MSC culture. In fact, Yew et al. and others have shown that hypoxic conditioning significantly improves MSC cell proliferation kinetics [10,11,12,13]. Therapeutic effects but also side effects of MSC on functionality [14], fibrosis [15], and the revascularization of cardiac tissue have been reported. Li and co-workers observed a reduced density of connexin 43 and an increased density of connexin 45 following cardiac remodeling, the imbalance of these membrane-bound molecules thereby affecting the physiological spread of electrical excitation. These effects decline after MSC treatment [8]. Despite advances in stem-cell therapy, the mechanisms underlying positive MSC treatment are still poorly understood. In our own recently published study, we examined arrhythmias in a new mouse model with re-induced ventricular arrhythmias. By performing a permanent second infarct seven days after a temporary first LAD ligation, the second intervention repeatedly reproduced ventricular arrhythmias [16]. In this new model, MSC therapy resulted in shorter QRS duration, shorter QTc intervals, and a reduced occurrence of VT in the early phase after the second intervention. The observed shortening of QRS points to the ability of MSCs to improve cardiac electrical conduction [16]. Boink and co-workers also observed a shortened QRS duration in a canine MI model after MSC transplantation [17]. However, antiarrhythmic behavior of human MSCs has also been shown in a clinical study following their intravenous injection after a reperfused MI [18].

In a recent study with patients after MI and coronary heart disease, we observed a preoperative biomarker signature of circulating stem cells in peripheral blood that correlated significantly with the postoperative response of myocardial regeneration [19]. Using RNA-seq, we found a distinct RNA gene expression pattern, which is characteristic of a reduced (non-responder) or increased (responder) proliferative reaction of bone marrow stem cells to angiogenesis [20].

Against this background, we have investigated the effects of intramyocardially injected hypoxic-preconditioned murine MSC on postinfarction arrhythmias in mice. Using subsequent RNA-seq of the affected infarct area, we studied the underlying mechanisms at the gene expression level in order to identify a unique RNA gene expression pattern, which is characteristic of the influence of MSC on the cardiac rhythm.

## 2. Results

### 2.1. MSC Functional Identification

**Figure 1:**
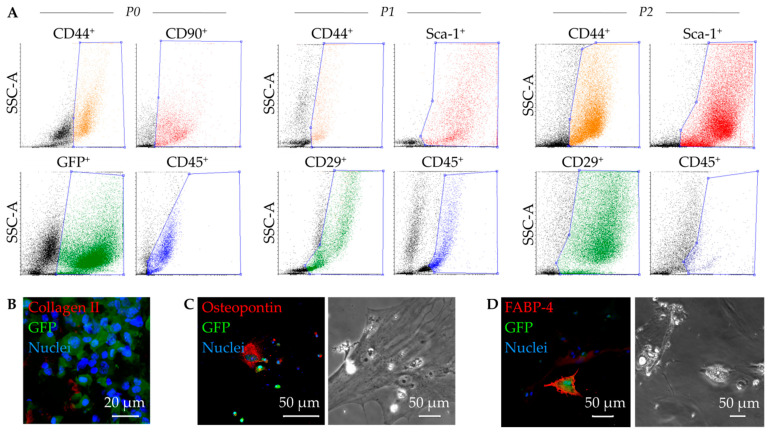
Functional characterization of murine mesenchymal stem cells (MSC). Representative dot plots of the flow cytometric analysis show GFP^+^ expressing cells (green, B6 eGFP mouse strain), as well as phenotypic alterations in expression of the MSC relevant surface markers over passages: CD44, CD90, CD45 at passage 0 (P0), as well as CD44, Sca-1, CD29, and CD45 at P1, and P2 (**A**). MSC demonstrate a multipotent differentiation potency proved by immunofluorescence staining and positive expression of collagen II after chondrocytic differentiation (**B**), osteopontin after osteocytic differentiation (**C**), and fatty acid binding protein 4 (FABP 4) after adipocytic differentiation (**D**). Phase contrast images illustrate morphological characteristics following respective differentiations: cytoskeletal structures for osteocytes (**C**) and lipid drops with visible vacuoles for adipocytes (**D**).

GFP^+^ MSC derived from murine bone marrow were cultivated under hypoxic conditions until second passage (Figure 1A). While the number of cells expressing the hematopoietic marker CD45 decreased over the cultivation period (5.2%), further flow cytometric analysis confirmed a mesenchymal phenotype, reflected by positive expression of the markers: CD44 (76.4%), CD29 (39.5%), and Sca-1 (90.3%).

In addition, we were able to show that the cultivated GFP^+^ MSC are capable of multipotent differentiation. In the assay performed, Collagen II^+^ was expressed in mature cartilage chondrocytes (Figure 1B), Osteopontin^+^ in osteocytes (Figure 1C), and FABP4^+^ in adipocytes with apparent formation of lipid droplets (Figure 1D).

### 2.2. Induced Ventricular Arrhythmias

**Figure 2:**
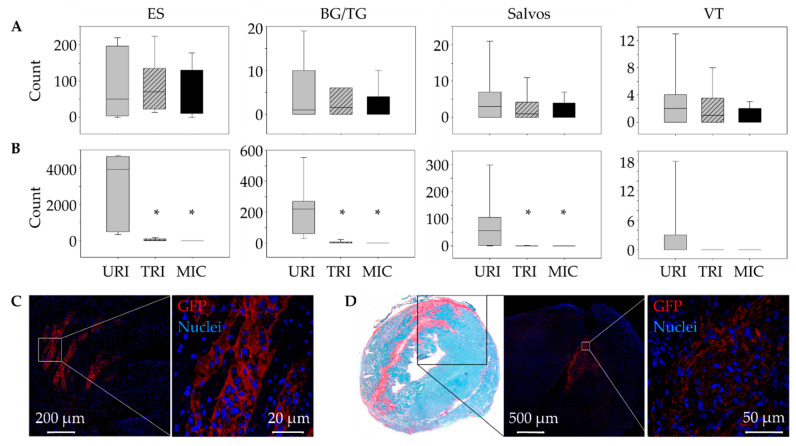
Antiarrhythmic effects of engrafted mesenchymal stem cells (MSC). Comparison of developed ventricular arrhythmias (VA) between 45 min and 12 h post ligation of the left anterior descending artery. While there were no significant differences in terms of the development of VA after the 1st infarction. (**A**), stem cell-treated animals showed significantly reduced VA after the 2nd infarction (**B**). Mean ± SD; * *p* ≤ 0.05 compared to URI (Mann–Whitney *U* Test). Detection of GFP^+^ MSC in heart tissue immediately after transplantation (**C**) and 9 days after the 1st infarction (**D**). ES, extrasystoles; BG/TG Bigemini/Trigemini; VT, ventricular tachycardias; URI, untreated re-infarction, *n* = 7; TRI, stem cell treated re-infarction, *n* = 6; MIC, myocardial infarction control, *n* = 7.

For transplantation of MSC, we used a mouse model, in which we re-induce ventricular arrhythmias one week after a first ischemia reperfusion by performing a second permanent ligation [16]. After the induction of the first infarction, a stem cell-treated animal and after the second infarction an untreated animal did not wake up again. As expected, no significant differences in arrhythmias were found between the groups after the first infarction (Figure 2A). In the time span up to 45 min following the second infarction also, no significant differences in arrhythmias were observed. From 45 min to 12 h, the URI group showed significantly more extrasystoles (ES), Salvos, and Bigemini (BG)/Trigemini (TG) compared to TRI. Ventricular tachycardias (VT) were not significantly increased (Figure 2B). However, VT only appeared in the URI group. Furthermore, arrhythmia reduction in TRI resulted in no significant differences compared to the MIC group.

After 9 days, the engrafted GFP^+^ cells were successfully detected on TRI mouse heart cryosections predominantly in the peri-infarct area by immunofluorescent staining (Figure 2C,D).

### 2.3. Alterations of the Infarct Scar

**Figure 3:**
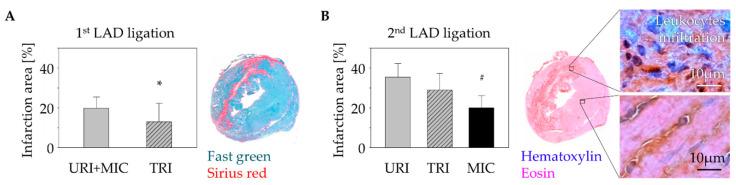
Change of myocardial infarct scar: Fast green/Sirius red staining of the murine heart cross sections 10 days after 1st LAD ligation showed a significant decrease of the infarction size in case of stem cell treatment (TRI) in contrast to untreated infarction (URI + MIC). Mean ± SD; * *p* = 0.05 (*t*-test); (**A**). Analysis of the leukocytes infiltration using Hematoxylin/Eosin staining 48 h after a 2nd LAD ligation in animals of URI and TRI group showed significant enlargement of the infarct scar area in contrast to infarction control (MIC) group. Mean ± SD; ^#^ *p* < 0.05 (*t*-test); (**B**).

Using fast-green Sirius-red staining, we were able to detect a significantly smaller infarct scar in animals with MSC therapy 9 days after the first infarction compared to animals without stem cell treatment (Figure 3A). Similar to our results in previously published studies on the influence of human MSC on arrhythmia after MI [16], there was no significant difference in leukocyte infiltration between animals with (TRI) and without (URI) stem cell treatment. However, only animals with a second infarction showed significantly higher leukocyte infiltration compared to animals without a second infarction (MIC; Figure 3B).

### 2.4. Identification of Antiarrhythmic Pathways and Associated Genes

**Figure 4:**
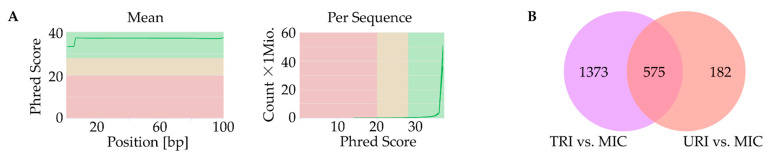
Identification of affected genes. Quality control with high Phred Score for all samples (green-good quality, yellow-mediocre quality, red-bad quality); (**A**); Venn-diagram with upregulated genes of TRI and URI compared to MIC as well as overlap of genes highly upregulated in TRI and URI (*q* ≤ 0.05); (**B**). URI, untreated re-infarction; TRI, stem cell treated re-infarction; MIC, MI control.

Next, we performed RNA-seq using samples from all mice (n = 20) to identify genes whose expression were affected by MSC treatment. As part of quality control, we found a high rate in mean quality sequencing scores represented by the Phred Score at a minimum of 30. Additionally, GC-Content per sequence was appropriate for mice at about 51.5%. Lastly, we checked quality scores per sequence and high Phred Scores were observed (Figure 4A).

In this study, we found significant changes between all investigated groups. Despite multi-level significance checks, about 2130 differences in gene expression were evident.

First, we compared the animal groups MIC vs. TRI, as well as MIC vs. URI. In this combination, nearly the same pathways with similar genes showed higher expression values. URI and TRI shared differences in pathways compared to MIC including Tyrobp casual network in microglia, type II interferon signaling, spinal cord injury, peptide GPCRs, microglia pathogen phagocytosis pathway, matrix metalloproteinase, macrophage markers, lung fibrosis, IL-5-signaling pathway, G1 to S cell cycle control, DNA replication, cytokines and inflammatory response, circulating monocytes and cardiac macrophage in diastolic dysfunction, chemokine signaling pathway and apoptosis. Besides common pathways, some genes are specific to one of the groups, while TRI showed more differently expressed genes then URI (Figure 4B).

**Figure 5:**
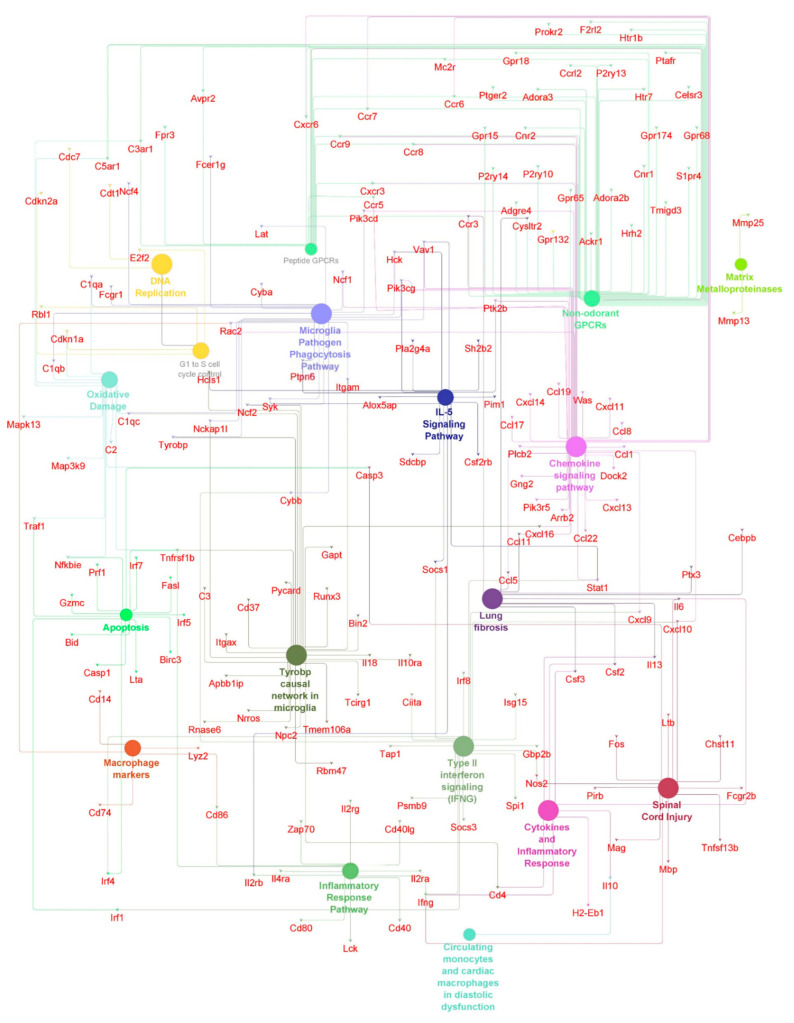
Antiarrhythmic pathways and associated genes in TRI-group. High expressed genes (small dots) and pathways (big dots) including macrophage markers, IL-5 signaling pathways, inflammatory and anti-inflammatory pathways (*q* ≤ 0.05). Node size-number of genes. Edges-relation based on kappa score 0.6 using experimental data.

**Figure 6:**
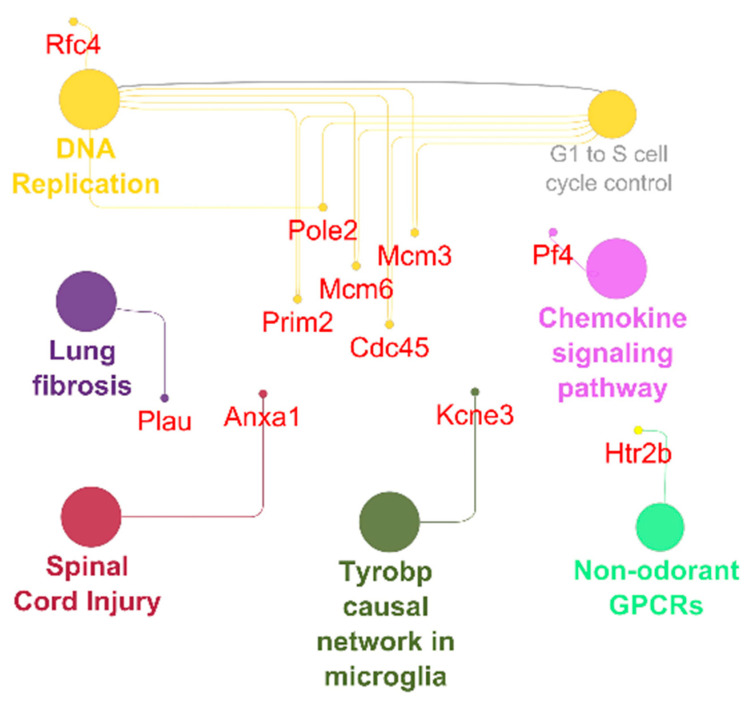
Antiarrhythmic pathways and associated genes in URI-group. High expressed genes (small dots) and pathways (big dots; *q* ≤ 0.05). Node size-number of genes. Edges-relation based on kappa score 0.6 using experimental data.

**Figure 7:**
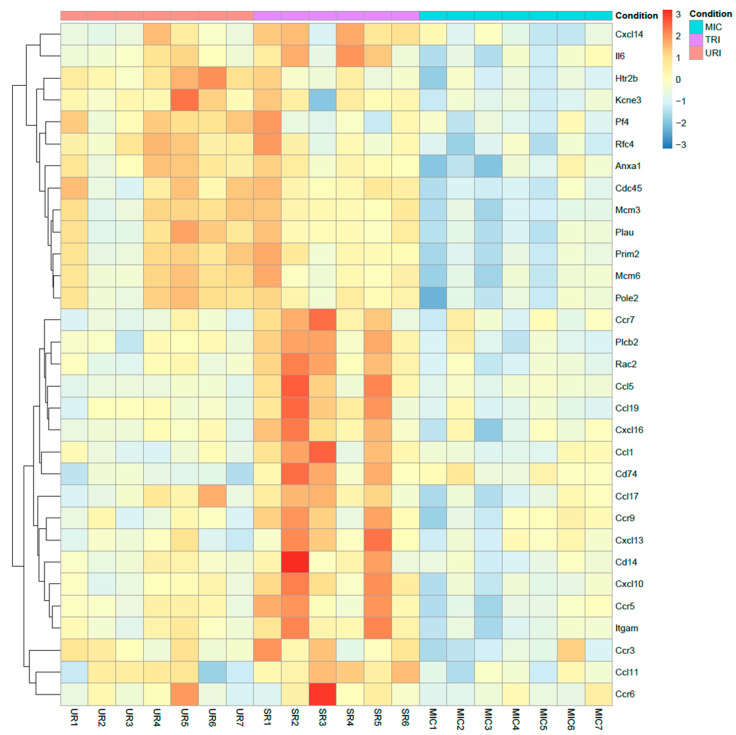
Heatmap of highly upregulated genes. TRI specific upregulated genes compared to MIC and URI using log_2_ (foldchange) values (*q* ≤ 0.05). Colors: Red-upregulated; yellow-neutral; blue-downregulated.

In the TRI-group, we saw higher pro- and anti-inflammatory genes including macrophage markers and IL-5 signaling pathway upregulation including genes such as CD14, CD74, Ccl1, Ccl5, Ccl11, Ccl17, Ccl19, Ccr5, Ccr7, Ccr9, Cxcl10, Cxcl13, Cxcl16, Il6, Itgam, Plcb2 and Rac2 (Figure 5). Furthermore, we observed pro-arrhythmic genes associated with heart failure and thrombosis after MI in URI predominantly Kcne3 and Pf4 (Figure 6). This indicates several interesting significantly differentially expressed genes specific for the treated and untreated group (Figure 7). In a nutshell, we see a high variety of genes highly expressed in treated animals showing both pro- and anti-inflammatory as well as pro- and anti-arrhythmic processes. In regard to the clinical aspects of this study, we found a reduced susceptibility of treated mice for simple and complex arrhythmia.

## 3. Discussion

This study showed the impact of hypoxic preconditioned murine MSC on arrhythmia in a post-infarction mouse model. While other studies use multipotent stem cells of human origin to investigate effects in immune-deficient mice, we followed a different approach for all anti-inflammatory and anti-arrhythmic effects to be considered. To this end, we used MSC isolated from B6-eGFP-mice, which were then implanted into C57BL/6J-mice. Based on our previous study, we utilized an infarction-re-infarction model to induce arrhythmia [16]. In our current study, we found a significant reduction of simple and complex arrhythmia after MSC transplantation. Interestingly, nearly all arrhythmia types, including ES, BG/TG, and salvos, in stem cell-treated animals with re-infarction (TRI) were similar to untreated animals without re-infarction (MIC).

Following a heart attack, acute inflammatory processes involving secretion of chemokines, growth factors and cytokines lead to destruction of the heart tissue and its subsequent remodeling with profound effects on cardiac electrophysiology. Although, there has been an improvement in the preventive and interventional therapy of MI, a 1-year mortality of 13% can be observed [21]. Furthermore, the post-infarct adaptations result in proarrhythmic tendencies in the context of myocardial remodeling [4] with a higher risk of malignant arrhythmias on the one hand and cardiac decompensation on the other [22]. However, cardiac function must be improved to further reduce mortality in the long term. For patients with MI, cell transplantation can be helpful and MSC have advantageous properties when used for cell therapy. In addition to easy handling and expansion in cell culture, little rejection in allogeneic and xenogeneic transplantation can be seen [23]. This immunomodulatory function enables a relatively simple donor-independent transplantation. Moreover, cardiac regeneration, neovascularization, anti-inflammatory properties, influences on contractility, remodeling, and post-infarct apoptosis have been described [6,7,24].

Following MSC implantation, pro-arrhythmic properties have been observed; therefore, these side effects cannot be excluded for MSC therapy [25]. While a differentiation of the MSC in the cardiac direction had been proven [26,27], no functional cardiomyocytes were found. In further studies, the electrophysiological properties of these cells showed major differences to physiological cardiomyocytes [8]. In an in vitro study by Chang and colleagues proarrhythmic properties were observed in the co-culture of MSC and neonatal myocytes [28]. In contrast, no proarrhythmic properties were observed in either extensive animal or clinical studies [18,29,30]. This discrepancy shows that there is still a need for research in this direction.

To measure more precisely the effects of syngeneic murine bone marrow MSC cell therapy, we purified and sequenced the heart tissue mRNA as well as observed the following significantly highly expressed TRI-specific genes in treated mice compared to untreated mice: CD14, CD74, Ccl1, Ccl5, Ccl11, Ccl17, Ccl19, Ccr5, Ccr7, Ccr9, Cxcl10, Cxcl13, Cxcl16, Il6, Itgam, Plcb2, Rac2 and URI-specific genes: Anxa1, Cdc45, Htr2b, Kcne3, Mcm3, Mcm6, Pf4, Plau, Pole2, Prim2, Rfc4. Most of the genes upregulated in TRI show protective functions, while a small subset are pro inflammatory genes (Figure 7). CD14 is one the characteristic markers of classical macrophages. Known polymorphism of CD14 showed susceptibility to MI in humans [31]. CD74, also known as MIF receptor, promotes cardiac stem cell survival proliferation and differentiation [32]. Inhibition of CCL1 promotes atherosclerosis and higher risk of cardiovascular events in mice [33]. CCL5 and CCR5 showed potential in angiogenesis with contribution to the recovery of cardiac function in infarcted heart [34]. Eotaxin mutation (CCL11) was found to elevate the risk of MI in patients without cardiovascular disease [35]. Additionally, CCR7 is involved in regulation of cardiac function in pericardial adipose tissue [36]. Furthermore, CXCL10 was found lowering cardiac fibrosis [37]. During the healing phase in the myocardium, mobilization of protective B-cells is activated by CXCL13, which is also upregulated in TRI [38]. On the other hand, IL-6 is known to aggravate inflammation. Single nucleotide polymorphisms of PLCB2 were linked to MI [39]. In contrast, CCL17 and CCL19 aggravates MI [40,41]. Furthermore, CCR9 is involved in arrhythmogenesis [42]. Lastly, RAC2 polymorphism is related to cardiotoxicity in breast cancer treatment, while the function of RAC2 in MI is not known yet [43]. URI specific genes showed mostly aggravating potential towards arrhythmogenesis and some protective functions (Figure 7). Anxa1 promoted an anti-inflammatory milieu by stimulating cardiac macrophages to release VEGF-A [44]. Upregulated in URI and downregulated in TRI, Kcne3 [45,46] seems to be linked to ventricular tachycardia in healed MI scar and Plated factor 4 is highly expressed in URI and downregulated in TRI promoting thrombosis after MI [47]. The functions of the following genes are either not conclusive or unknown: Cdc45, Htr2b, Mcm3, Mcm6, Plau, Pole2, Prim2, Rfc4.

In addition to the already known anti-inflammatory pathways after MI and stem cell therapy, for the first time, we observed reductions in simple and complex arrhythmias, a higher variety of pathways, as well as several protective functions. All highly expressed protective genes in animals that had received stem-cell therapy were found to be either lowering the risk of heart failure, cardiomyopathy or arrhythmogenesis. The absence of these specific genes has been associated with higher arrhythmia rates and even key mutations for previously described arrhythmic disorders. In conclusion, our intervention with preconditioned murine MSC after MI led to a reduction in cardiac arrhythmia and revealed gene regulation pathways that may have direct influence on arrhythmogenesis.

## 4. Materials and Methods

### 4.1. MSC Isolation and Cultivation

For quantitative coverage of all cell transplants, we used a total of two 10-week-old B6 eGFP donor mice (Jackson Laboratory, Maine, ME, USA) from the same birth to isolate murine bone marrow-derived mononuclear cells. Mice were killed by dislocating the cervical spine. After the tibiae and femora had been dissected out, they were transferred to a Petri dish with 5 mL MesenCult™ expansion kit (supplemented with MesenCult 1:10 and MesenPure, 1:1000; STEMCELL Technologies, Vancouver, BC, Canada). After removal of the epiphyses, 2.5 mL of MesenCult medium were injected into the open medullary canal using an 18 gauge injection syringe (B. Braun, Melsungen, Germany). Cells were filtered with a 70 µm EASYstrainer (Greiner Bio-One, Frickenhausen, Germany), centrifuged at 300× *g* and room temperature for 10 min and cultured with a density of ~6 × 10^3^ per cm^2^ at 37 °C, 5% CO_2_ and 1% O_2_ for 5 days. When ~90% confluence was reached, cells were passaged using 1% Trypsin/EDTA (PAN Biotech, Aidenbach, Germany) for 2 min at 37 °C and 5% CO_2_. After reaching 90% of confluency, cells at passage 0, 1 and 2 were used for the follow-up experiments.

### 4.2. Flow Cytometric Analysis

The mesenchymal pattern [48] was verified by flow cytometry analysis. All antibodies were based on rat anti-mouse, while appropriate rat isotypes for control of gating strategy were considered. The following antibodies were employed: anti-CD105-phycoerythrin (PE) (1:10, Miltenyi Biotec GmbH, Bergisch Gladbach, Germany), anti-CD29-Fluorescein (FITC, 1:20), anti-CD44-eFluor 450 (1:80, Pacific Blue, both from eBioscience Inc. San Diego, CA, USA), anti-CD45-Peridinin-chlorophyll-protein complex (PerCP, 1:50), anti- Ly-6A/E (Sca-1)-PE-Cyanin 7 (PE-Cy7, 1:20) (both from Becton Dickinson (BD) Biosciences, Heidelberg, Germany), as well as a primary antibody: goat polyclonal Thy-1 (CD90) (1:50, Santa Cruz Biotechnology, Inc., Dallas, TX, USA). To distinguish living from dead cells, Zombie Aqua™ Fixable Viability Kit was applied (BioLegend, San Diego, CA, USA).

Firstly, mononuclear cells obtained at passage 0, 1 and 2 were resuspended in phosphate buffered saline (PBS) and stained with Zombie Aqua™ dye for 15 min at room temperature. Following a wash step in PBS, cells were suspended in cold MACS^®^ buffer containing PBS, 2 mM EDTA, 0.5% bovine serum albumin and FcR mouse blocking reagent (1:10, Miltenyi Biotec). Antibodies were incubated for 30 min in the dark at 4 °C, washed once with PBS. For the sample with Ab against CD90, cells were processed for an additional incubation step (30 min) with a secondary antibody donkey anti-goat allophycocyanin (APC) (1:300, R&D Systems, Minneapolis, MN, USA). Finally, probes were acquired on BD^TM^ LSRII flow cytometer and analyzed using FACS Diva software, version 6.1.2 (both BD). Values were expressed in % and generated from the region of the viable singlets.

### 4.3. Proof of the Differentiation Potential

In order to assess the differentiation potential of murine MSC, cells of passage 2 were cultivated in either osteogenic, adipogenic or chondrogenic media provided in the Mouse Mesenchymal Stem Cell Functional Identification Kit (SC010; R&D Systems, Wiesbaden, Germany) according to the manufacturer’s instructions. For adipogenic, osteogenic, and chondrogenic differentiation, cells were seeded at a density of 2.1 × 10^4^ cells/cm^2^ on uncoated glass coverslips in 24-well plates, 4.2 × 10^3^ cells/cm^2^ on fibronectin-coated (10 µg/mL; Millipore, Darmstadt, Germany) glass coverslips in 24-well plates, and 2.5 × 10^5^ cells pelleted in conical 15 mL falcon tubes, respectively. Following cultivation (at average time 21 days), adipocytes and osteocytes were fixed with 4%—buffered formaldehyde at room temperature before immunofluorescence staining (IF). Chondrocyte pellets were fixed and snap-frozen in O.C.T. Compound Tissue-Tek^®^ (Sakura Finetek, Japan) in liquid nitrogen, followed by generating 5 µm-thick cryosections. Primary antibodies provided in the kit were applied for: adipocyte (goat anti-fatty acid binding protein 4, FABP4), osteocyte (goat anti-osteopontin), and chondrocyte (sheep anti-collagen-II) detection. Respective secondary antibodies for adipocyte/osteocyte donkey anti-goat Alexa-Fluor^®^-568 (Cat. No. A11057) and for chondrocyte donkey anti-sheep Alexa-Fluor^®^-633 (Cat. No. A21100; all Thermo Fisher Scientific, Waltham, MA, USA) were applied. Nuclei were counterstained with DAPI (Sigma Aldrich, Taufkirchen, Germany). Images were taken using an ELYRA PS.1 LSM 780 microscope and the ZEN2011 software (both Carl Zeiss Jena GmbH, Jena, Germany).

### 4.4. Animals

All animal procedures conform to the guidelines from Directive 2010/63/EU of the European Parliament on the protection of animals used for scientific purposes. The federal animal care committee of LALLF Mecklenburg-Vorpommern (Germany) approved the study protocol (approval number LALLF M-V/TSD/7221.3-1-037/16).

### 4.5. Ambulatory ECG Monitoring and Ligation of the Left Anterior Descending Artery

For ambulatory electrocardiogram (ECG) monitoring, a telemetric device (Data Sciences International, DSI, St. Paul, MN, USA) was implanted in all 12 to 14 week-old female C57BL/6J mice (Charles River, Germany; *n* = 22). For this purpose, all mice were anesthetized using 50 mg/kg Narcoren^®^ injected intra-peritoneally (Boehringer Ingelheim Vetmedica, Germany). Afterwards a median longitudinal incision was made on the back along the ribs to insert a telemetric transmitter (TA11ETA-F10 Implant; DSI) into a subcutaneous pocket at the level of the lower back with paired wire electrodes placed over the thorax with leads tunneled to the right upper shoulder and left lateral lower thorax. Using Ponemah Physiology Platform (DSI) Heart rate, PR interval, and QRS-complex were recorded in a time span of 48 h after myocardial infarction. Telemetric ECG signal was qualitatively and quantitatively analyzed using ECG auto 1.5.11.26 software (EMKA Technologies, Paris, France).

To induce MI, mice were randomly assigned into three groups: untreated re-infarction (URI), stem-cell treated re-infarction (TRI), and MI control (MIC) [16]. Animals of all three groups underwent 1st thoracotomy, as well as the ligation of the left anterior descending (LAD) artery. After 45 min, each mouse received an intra-myocardial application of 20 µL BD Matrigel^TM^ Matrix (Thermo Fisher Scientific Waltham, MA, USA)/MACS^®^ buffer in a ratio of 1:2 in the form of four injections of 5 µL each along the border of the blanched myocardium. For stem-cell treatment (TRI group) the application solution contained 400,000 MSC. Subsequently, the node was re-opened (ischemia/reperfusion) leaving the ligature in the heart looped around the LAD with their ends kept inserted in a soft flexible subcutaneously placed tube.

After the observation period of seven days, every mouse underwent a 2nd thoracotomy. Additionally, animals of the URI and TRI group underwent permanent LAD ligation at the same site using the suture from the 1st ligation (re-infarction). The rubber tube has facilitated and eased the finding of the suture material. MIC operated mice underwent identical 2nd surgical procedure without LAD-ligation.

### 4.6. Basic ECG Parameter

To evaluate the antiarrhythmic effects of MSC-transplantation, we measured the quantity of extrasystole (ES), Salvo, bigemini/trigemini (BG/TG), and ventricular tachycardia (VT) manually. Marking every instance of arrhythmic events using ecgAUTO v3.3.0.28 software. The total was aligned into four timespans: 0–15 min, 15–45 min, 45 min–12 h, and >12 h, respectively.

### 4.7. Organ Harvesting and Tissue Processing

Each mouse was euthanized by cervical dislocation 9 days after the 1st infarction. Hearts were removed, embedded in O.C.T.^TM^ Compound (Tissue-Tek^®^; Alphen aan den Rijn, The Netherlands), and snap-frozen in liquid nitrogen. The infarction area was divided into four horizontal planes starting from the apex to the base. These regions where cut into 5µm cryosections and intermediate cuts. The cryosections and tissue from intermediate layers were stored at −80 °C for the further subsequent experiments.

### 4.8. GFP^+^ Cells Detection

Rabbit anti-GFP primary antibody (Abcam, Trumpington, Cambridge, UK) was used for detecting GFP^+^ cells on mouse heart cryosections. Donkey-anti-rabbit-Alexa-Fluor^®^-568 (Cat. No. A10042) served as conjugated secondary antibody followed by counterstaining with DAPI (both from Molecular Probes^TM^, Thermo Fisher Scientific).

### 4.9. Infarction Size and Leukocytes Infiltration Area Analysis

Randomly chosen histological heart sections of four horizontal infarct levels were stained with Fast Green FCF (Sigma-Aldrich, Saint Louis, MO, USA) and Sirius Red (Chroma Waldeck GmbH & Co. KG, Münster, Germany) assessing tissue localization and distribution of connective fibers. Two contiguous levels of the heart which represent the major infarction ratio were quantitatively estimated using computer aided image analysis (AxioVision LE Rel.4.5 software, Carl Zeiss AG, Oberkochen, Germany). To evaluate leukocytes infiltration area 48 h after the 2nd ligation, the two contiguous levels of the heart which represent the major infarct ratio were stained with Hematoxylin (Merck, Darmstadt, Germany) and Eosin (Thermo Shandon Ltd., Runcorn, UK) and representative images were taken using computerized planimetry (AxioVision LE Rel.4.5 software).

### 4.10. RNA-Sequencing

Heart tissue from the intermediate layers of all mice (*n* = 20) were lyzed in TRIZOL^®^ reagent (Thermo Fisher Scientific). RNA was isolated following the instructions of the TRIZOL^®^ standard protocol. Second, isolated RNA was precipitated with 2.5 volumes ethanol under high salt conditions (10% of 3M sodium acetate, pH 5.2). After DNase digest (Thermo Scientific) the RNA was finally purified using Agencourt RNAClean XP beads (Beckman Coulter, Indianapolis, IN, USA). Purified RNA was analyzed using Bionalyzer (Agilent Technologies, Santa Clara, CA, USA) using RNA 6.000 Nano Chips (Agilent). Quality tested RNA was used to construct sequencing libraries using the Universal Plus mRNA–Seq Technology (NuGEN Technologies, Inc., CA, USA) according to manufacturer’s instructions. Briefly, mRNA was selected by oligo d(T) beads, reverse transcribed, and cDNA from Globin messengers was removed by the Globin depletion module (NuGEN). Quality controlled and quantified libraries were sequenced on a HiSeq1500 system (Illumina, Inc., San Diego, CA, USA) in single-end mode (100 nt read length).

RNA-seq data analysis was performed using Galaxy (accessed on: 1 August 2022; https://usegalaxy.eu/). First, data was cleaned using Trim Galore to remove used adapters. Utilizing FastQC and MultiQC, we analyzed the mean quality score, GC content per sequence, and quality per sequence. Closing Quality Control, we analyzed high expression genes using *q* < 0.05 with Benjamini–Hochberg-correction and log_2_ (fold change) of ≥|1|. For alignment RNA-Star (Galaxy Version 2.6.0b-1) alongside the most recent *mus musculus* genome file (Mus_Musculus mm10, UCSC) were utilized. Additionally, FeatureCounts (Galaxy Version 1.6.3) was used to determine the quantity of genes expressed. DESeq2 (Galaxy Version 2.11.40.4) filtered significantly differentially expressed transcripts for further downstream analysis (*q*-value < 0.05, log_2_ fold change of ≥|1|).

Lastly over- and under-expressed genes were visualized and compared between all groups. Using a *p*-value of <0.05 adjusted for multiple testing with the Benjamini–Hochberg procedure, significant changes in expression per group using Cytoscape 3.8, ClueGo with WikiPathways as pathway source, were identified. In this step a *q*-value (<0.05) with Bonferroni stepdown and Kappa-Score of 0.6 were used. Afterwards networks containing all significantly differentially expressed genes with log_2_ fold change >|1| were created.

### 4.11. Statistical Analysis

Statistical analysis was performed using SigmaStat 3.5 (U.S.) and IBM SPSS Statistics for Windows (Version 22.0., IBM Corp., Armonk, NY, USA). Comparisons of two experimental groups were performed after Shapiro–Wilk-Test using in this data the Mann–Whitney *U* test. *p* values ≤ 0.05 were considered as statistically significant.

## Data Availability

https://usegalaxy.eu/u/ahmad/h/clean (accessed on 4 August 2022).
